# Immune system and microbiome in the esophagus: implications for understanding inflammatory diseases

**DOI:** 10.1111/febs.16103

**Published:** 2021-07-13

**Authors:** Tanay Kaymak, Petr Hruz, Jan Hendrik Niess

**Affiliations:** ^1^ Department of Biomedicine University of Basel Switzerland; ^2^ Clarunis ‐ University Center for Gastrointestinal and Liver Diseases Basel Switzerland

**Keywords:** dendritic cells, eosinophilic esophagitis, esophagus, microbiome, mucosal immunity

## Abstract

The gastrointestinal tract is the largest compartment of the body's immune system exposed to microorganisms, structural components and metabolites, antigens derived from the diet, and pathogens. Most studies have focused on immune responses in the stomach, the small intestine, and the colon, but the esophagus has remained an understudied anatomic immune segment. Here, we discuss the esophagus' anatomical and physiological distinctions that may account for inflammatory esophageal diseases.

## Graphical abstract

Esophageal inflammatory diseases develop in a genetic‐predisposed individual with an impaired relationship between the epithelium, the immune system, and the microbiome. Here, we review the mucosal immune system, microbiota, and epithelium in health and disease with implications for inflammatory conditions in the esophagus.

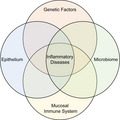

**Abbreviations**
DC: dendritic cells
EAC: esophageal adenocarcinoma
EoE: eosinophilic esophagitis
GERD: gastroesophageal reflux disease
ILC: innate lymphoid cells
LC: Langerhans cells
PPI: proton pump inhibitors

## Introduction

The esophagus, besides the transport function of food, is an organ critical for mucosal immunity. There is an increasing awareness that antigens and metabolites derived from microorganisms and the diet are in contact with the esophagus' mucosal immune system. Food proteins are digested and proteolyzed by the stomach's low pH and proteases secreted by the pancreas after they have passed the esophagus. These findings have led to skyrocketing numbers of studies investigating immune responses in the small and large intestine. However, the esophagus remains a somewhat understudied immune organ, although it is easily accessible by endoscopy without specific preparations and allows biopsy sampling more straightforward than other parts of the human intestine.

Eosinophilic esophagitis (EoE) can clinically present with food impaction or dysphagia histologically characterized by an eosinophil‐predominant inflammation (> 15 eosinophils per high power field) [1]. Worldwide, EoE has an increasing incidence and prevalence, with currently an incidence of 4.4–7.4 per 100 000 individuals per year and a prevalence of 43 per 100 000 individuals [2]. It has been shown that due to an impaired esophageal epithelial barrier, dietary antigens, structural components of microbes, and bacterial microorganisms penetrate the esophageal mucosa. The recognition of these dietary antigens or bacterial metabolites by esophageal epithelial cells and underlying immune cells leads to an inflammation characterized by increased expression of Th2 cytokines, such as thymic stromal lymphopoietin (TSLP), IL‐5, and IL‐13 [3, 4, 5, 6, 7]. However, the cellular composition of the mucosal immune system in the esophagus is primarily unknown.

Moreover, studies have recently appreciated that the esophagus has its distinct microbiome of predominantly gram‐positive bacteria dominated by *Streptococcus* [8, 9, 10]. The microbiome's dynamic changes depending on the ingestion of meals, nocturnal rhythms, and diseases are beyond this review's scope. However, they have been anticipated as a prerequisite for changes in immune cells' cellular composition in health and disease.

With few exceptions, most reviews discussing the mucosal immune system's structure focus on the small intestine and colon. Considering that the esophagus' immune‐mediated diseases have an increasing incidence and prevalence, in this review, we discuss the esophagus's immune system's organization and point out the specifics of the esophagus' immune system.

## The anatomic structure of the esophagus

The esophagus is a continuous fibromuscular tube composed of the epithelium, lamina propria, submucosa, muscle layers, connective tissues, and the adventitia. It passes from the hypopharynx behind the trachea and heart through the mediastinum and diaphragm into the stomach. The esophagus is approximately 20–27 cm long, and its explicit function is the transportation of the alimentary bolus from the mouth into the stomach. However, the esophagus is not only a transit organ and also has a critical function in the mucosal immune response. At least three layers of squamous cells, the stratified squamous epithelium, line the esophagus' luminal side. The esophagus and the oral cavity represent the upper gastrointestinal tract (GIT) and belong, together with the vaginal cavity, to the type 2 mucosal surface lined by stratified squamous epithelium, primarily serving as a physical protective barrier. The mucosal surface of the esophagus lacks mucosa‐associated lymphoid tissues, the polymeric immunoglobulin receptor, goblet cells, and Paneth cells in contrast to type 1 mucosal surfaces (e.g., lower intestine, lungs, and uterus) lined by a single layer columnar epithelium. As a consequence, the esophagus lacks a thick mucus layer and IgA. However, one study investigating the presence of immunoglobulins in the esophagus of HIV‐infected individuals has described IgA as the predominant immunoglobulin in the esophagus [11]. Nevertheless, it remains unclear whether, in immunodeficient individuals, IgA is secreted across the epithelium in the esophageal lumen or derived from IgA‐containing saliva.

It is somewhat surprising that, in contrast to the colon, the esophagus lacks a thick mucus layer, despite the presence of hydrogen ions (H^+^) and bile acids from the stomach and duodenum. Esophageal peristalsis and gravity in an upright position clear 95% of refluxed acid, but ~ 5% of the refluxed stomach contents remain in the esophagus [12]. The esophagus has to neutralize hydrogen ions and bile acids, facilitated by bicarbonate, antimicrobial peptides, and lactoferrin containing saliva forming a soluble mucus capable of lubrication to protect the epithelium [13, 14]. The unprotected esophageal epithelium is susceptible to contact with food contents, hydrogen ions, and bile acids. After entry of luminal content through the disrupted epithelial barrier into the esophagus, immune cells beneath the epithelium initiate a specific defense mechanism to protect the esophagus.

## The architecture of the squamous epithelium as a first defense line

The esophageal squamous epithelium consists of three distinctive layers: the stratum corneum (superficial layer), stratum spinosum, and stratum germinativum (basal cell layer) [15]. Each layer plays its part in maintaining epithelial integrity to protect the underlying tissue from environmental products and prevent the development of esophageal pathologies. The glycocalyx covers the esophageal epithelium's apical membrane providing a robust physical barrier that shields the esophagus from damage. In the stratum corneum, filaggrin connects with intermediate keratin filaments creating a lipid‐protein matrix that forms an impenetrable epithelial barrier and an intercellular glycocalyx [16]. Moreover, intercellular junctional complexes consisting of tight and adherens junctions intertwine individual epithelial cells of the stratum spinosum, limiting the flux of molecules, ions, and acid into the intercellular space, thereby protecting the epithelium from damage. Tight junctions interconnecting cell membranes and regulating paracellular ion‐permeability are at the apical side of the epithelium. Proteins such as claudin, occludin, zonulin, and junctional adhesion molecules span over the intercellular space and connect with neighboring cells' cytoskeleton [15]. Dysfunctional tight junctions lead to an impaired epithelial barrier facilitating electrolyte and fluid loss and increasing susceptibility to atopic diseases (e.g., atopic dermatitis, asthma, and EoE). Malfunction of the tight junction proteins (e.g., claudin and occludin) can occur in these diseases [5, 17, 18, 19]. Another crucial functional structure represents the adherens junction consisting of the transmembrane protein E‐cadherin and the intracellular catenins and vinculin, which ensure attachment to actin filaments. Adherens junctions are responsible for stabilizing cell‐cell adhesion, regulating the actin cytoskeleton, and mediating intracellular signaling and transcription regulation [20]. The apical junction complex's most basal structure is the desmosomes that consist of two transmembrane proteins, desmocollin, and desmoglein. Desmosomes support the adherens junctions in cell‐cell adhesion [21]. The junctional complex formation is calcium‐dependent [22], as paracellular permeability increases, and transepithelial resistance decreases after the experimental removal of calcium in cell cultures. Consequently, esophageal stem cells isolated from biopsies have to be cultured in a calcium‐rich medium to differentiate and generate esophageal organoids [23].

When noxious agents overcome pre‐epithelial and epithelial defenses, postepithelial defense mechanisms are in place to prevent tissue destruction. Paracellular glycoconjugates with buffer capacity [24] and the mucosal blood supply, which not only provides oxygen, nutrients, and bicarbonate ions but also removes metabolic byproducts, such as hydrogen ions, lactic acid, and CO_2_, have protective functions for the esophageal epithelium [14, 25, 26]. The epithelium can renew and repair wounds after damage. Restitution is a quick repair in minutes to hours by migrating adjacent viable cells to replace dead cells and close wounds [27]. The second form of epithelial repair replaces dead cells with newly generated viable cells by mitosis, a more protracted process of days to weeks [28]. Furthermore, immune cells located beneath the epithelium serve as an additional firewall to remove incoming antigens and pathogens after a barrier breach has occurred.

## Acid‐induced esophagitis

When the esophagus fails to clear the reflux of gastric content and bile acids, gastroesophageal reflux disease (GERD) can develop. In the general population, 10–30% of adults suffer from heartburn, retrosternal pain, and regurgitation [29]. Obesity, hiatal hernia, delayed gastric emptying, and chronic pulmonary disease can increase thoracoabdominal pressure gradients and predispose to GERD [30, 31, 32, 33, 34, 35]. GERD complications include erosive esophagitis, peptic strictures, Barrett's esophagus with the replacement of the esophageal squamous epithelium by intestinal columnar epithelia with sequential development of dysplasia, and esophageal carcinoma [29, 36, 37, 38]. Despite symptomatic reflux, up to 70% of GERD individuals do not develop esophageal erosions [39]. Microscopically, dilated intercellular spaces are also present in nonerosive reflux disease [40, 41]. It has been suggested that chronic acid exposure induces epithelial cells to secrete pro‐inflammatory cytokines resulting in a mucosal immune cell infiltrate dominated by neutrophils and eosinophils, causing erosions [42]. This hypothesis gives a potential explanation as why macroscopic changes are absent in nonerosive reflux characterized by potentially lower acid exposure, mostly preserved esophageal clearance, and the absence of a pro‐inflammatory cytokine signature [42, 43, 44]. These studies received further support by data that observed significant immune cell infiltration only in erosive reflux disease, despite the observation that erosive and nonerosive reflux disease both presented with microscopic changes, such as dilated intercellular spaces [38, 45]. The pro‐inflammatory cytokine pattern in erosive reflux disease includes the neutrophil chemoattractants IL‐1β and IL‐8, and IL‐6 and chemokines, such as RANTES, MCP‐1, MIP1‐a, platelet‐activating factor, and the eotaxins [46, 47, 48]. Although neutrophils dominate the immune cell infiltrate in GERD [49], eosinophils accumulate in up to 50% of GERD posing a clinical challenge for the distinction from EoE [49]. Since proton pump inhibitors (PPIs) have proven to be an effective treatment for GERD without overt side effects, immunological research in the esophagus has shifted from GERD to EoE, an immune‐mediated disease induced by food allergens and with a treatment spectrum of limited efficacy.

## Immune cells as critical firewalls in the esophagus

A unique microbiome, mainly gram‐positive bacteria, colonizes the esophagus, formerly thought to be only transiently populated by swallowed bacteria from the oral cavity. When these potentially harmful microorganisms and metabolites pass the squamous epithelium, the immune system beneath the epithelium has to fight these threats and defend the host. Only a few immune cells are present in the esophagus of healthy individuals, but increase during infection or inflammation. It needs to be pointed out that most studies have so far investigated the distribution of immune cells in the esophagus, but functional studies describing immune cells in esophageal diseases are scarce. Comparable to the skin, CD1^+^ Langerhans cells (LCs) are present in the esophageal epithelium. As in the skin, LCs in the esophageal epithelium are in close association with lymphocytes in the suprabasal layer [50]. Access to esophageal tissue is only possible by taking biopsies from the esophagus during invasive esophagogastroscopy, in contrast to noninvasive biopsy collection from the skin and the oral cavity. Studies by Novak *et al*. have investigated mucosal dendritic cells (DCs) in the oral cavity [51, 52]. These studies allow some assumptions on esophageal DCs' characteristics because of the oral cavity's proximity to the esophagus. Furthermore, the DCs in the oral cavity reside beneath the squamous epithelium like in the esophagus [53]. In contrast to the small and large intestine, the esophagus lacks organized secondary lymphoid tissues with follicle‐associated epithelium, where antigens are sampled and presented to T cells. In the esophagus, DCs phagocytose antigens directly from the lumen [54]. It has been suggested that DCs induce a tolerogenic state by presenting antigens to T cells in the epithelium, the lamina propria, or after migration to regional lymph nodes [53, 55]. During inflammation, blood‐borne DCs infiltrate, in addition to resident LCs, the esophageal epithelium, take up antigens, and migrate to the mediastinal lymph nodes to induce an immune response [55, 56]. Comparable to the small intestine, tolerance‐inducing DCs stay in an immature state and do not express CCR7, which binds CCL19 and CCL21, two cytokines responsible for the homing of immune cells to draining lymph nodes, whereas infiltrating DCs fully differentiate and begin to express CCR7 [55]. Higher expression of T‐cell inhibitory surface molecules (e.g., B7‐H) in tissue‐resident mucosal DCs compared to epidermal DCs reflects the specific esophageal tissue environment [51].

After antigen presentation by DCs to T cells in the regional lymph nodes, the effector T cells circulate back to the esophagus. Under noninflamed conditions, only a few T cells are present in the esophageal epithelium, comprising mainly CD8^+^ T cells like the intraepithelial lymphocyte compartment in the small intestine [57]. During inflammation, the T cell numbers increase and can form dense clusters of CD4^+^ and CD8^+^ T cells reminding of isolated lymphoid follicles in suprabasal regions of the esophagus [53]. Although T cells are widely present in the gut and lung, T cells' characterization in the esophagus is lacking. It is of interest to determine the distribution of IFN‐y‐producing Th1, IL‐13‐producing Th2, and IL‐17A‐producing Th17 cells in the esophagus of healthy individuals and to compare them to T cells in the inflamed esophagus. There have also been innate lymphoid cells (ILCs) described in the esophagus. Doherty *et al*. have reported the presence of ILC2s in the human esophagus that increased in numbers in EoE and correlated with the degree of mucosal eosinophilia. The stimulation with IL‐2, IL‐33, and TSLP, three cytokines with increased expression in EoE, expanded the numbers of ILC2s *in vitro* [58]. To this end, we did not find any studies describing the presence of type 1 and type 3 ILCs in the esophagus.

Taken together, immune cells in the esophagus serve as critical firewalls in the esophagus. However, there is a great need to better phenotype and functionally describe the distribution of immune cells in the esophagus and how these cells' composition changes in esophageal diseases.

## The esophageal microbiome

In recent years, it became apparent that the microbiome is essential for the proper functioning of the GIT, the immune system, defense against pathogens, metabolism, and energy regulation. Approximately 10% of all metabolites in the peripheral blood stem from the microbiota [59]. Our microbiota provides roughly 10% of our daily ingested calories due to fermentation [60]. The microbiota also protects from infectious esophagitis, such as Candida esophagitis with the Candida albicans serotype, the most common Candida species causing esophagitis [61]. The microbiome interferes with the immune system, and its critical relevance for the digestive system and metabolism is one of the most popular current research topics. While the small intestine and colon microbiota are at the center of research, the esophageal microbiome has been widely neglected. It has been assumed that the esophageal microbiome does not exist and only reflects a transient bacterial mix of swallowed oral and refluxed gastric commensals. Methodical limitations further hampered the esophageal microbiome research to acquire samples noninvasively and without oropharyngeal cross‐contamination [62].

Nonetheless, recent studies revealed an independent resident microbiome in the esophagus [63, 64, 65]. Indeed, several phyla found in the oral cavity and lungs are also present in the esophagus (e.g., *Firmicutes*, *Fusobacteria*), suggesting that swallowed oral commensals influence the formation of the resident esophageal microbiota (Table 1). The absence of several oral microbiota strains in the esophagus indicates that not all bacteria present in the oral cavity can colonize and survive in the esophagus [63, 64, 65, 66, 67]. Moreover, the esophageal mucosa is populated with a unique microbiota as some Firmicutes members, including *Clostridium*, *Eubacterium*, *Megasphaera*, *Mogibacterium,* and *Moryella* populate only the esophagus and not the oral cavity [8, 9, 10]. Approximately 140 bacterial species have been identified by 16s rRNA‐sequencing in the esophagus that constitutes the esophageal microbiome, with the most common bacteria belonging to six different phyla (70% Firmicutes, 20% Bacteroidetes, 4% Actinobacteria, 2% Proteobacteria, 2% Fusobacteria, and 1% TM7) [63, 64, 65]. The dominant genera characterizing the esophageal core microbiome are *Streptococcus*, *Prevotella*, *Veillonella,* and *Fusobacterium* [8, 9, 10]. The esophageal core microbiome's composition presents with variations along the esophagus but persists between gender and age groups [62, 64, 65, 68].

**Table 1:** The esophagus's core microbiome and alteration in EoE and GERD. + present; ++ abundant; +++ highly abundant; C, Core microbiome; ↑ increased abundance; ↓ reduced abundance; N.d., not determined.

Phylum	Healthy	EoE^a^	GERD^a^
Firmicutes	+++ Harris *et al*. [74], Pei *et al*. [63], Fillon *et al*. [64]	↓ Harris *et al*. [74]	↑ Harris *et al*. [74], ↓ Liu *et al*. [73]
*Veillonella*	C, Harris *et al*. [74], Fillon *et al*. [64]	↓ Harris *et al*. [74]	↑ Harris *et al*. [74]
*Streptococcus*	C, Harris *et al*. [74], Fillon *et al*. [64], Norder Grusell *et al*. [66]	↓ Harris *et al*. [74]	↑ Harris *et al*. [74]
Bacteriodetes	++ Harris *et al*. [74], Pei *et al*. [63], Fillon *et al*. [64]	↔ Harris *et al*. [74]	↓ Harris *et al*. [74], ↔ Liu *et al*. [73]
*Prevotella*	C, Harris *et al*. [74], Fillon *et al*. [64], Norder Grusell *et al*. [66]	↔ Harris *et al*. [74]	↓ Harris *et al*. [74]
Actinobacteria	+ Pei *et al*. [63], Fillon *et al*. [64]	N.d.	N.d.
*Corynebacterium*	+ Benitez *et al*. [65]	↑ Benitez *et al*. [65]	N.d.
Proteobacteria	+ Harris *et al*. [74], Pei *et al*. [63], Fillon *et al*. [64]	↑ Harris *et al*. [74]	↓ Harris *et al*. [74], Liu *et al*. [73]
*Haemophilus*	+ Fillon *et al*. [64], Norder Grusell *et al*. [66]	↑ Harris *et al*. [74]	↓ Harris *et al*. [74]
*Campylobacter*	+ Benitez *et al*. [65]	↑ Benitez *et al*. [65]	N.d.
*Neisseria*	+ Fillon *et al*. [64], Norder Grusell *et al*. [66]	↑ Harris *et al*. [74], Benitez *et al*. [65]	↓ Harris *et al*. [74]
Fusobacteria	+ Harris *et al*. [74], Pei *et al*. [63], Fillon *et al*. [64]	↓ Harris *et al*. [74]	↓ Harris *et al*. [74], ↑ Liu *et al*. [73]
*Fusobacterium*	C, Harris *et al*. [74], Fillon *et al*. [64], Norder Grusell *et al*. [66]	↓ Harris *et al*. [74]	↓ Harris *et al*. [74]
TM7	+ Pei *et al*. [63], Fillon *et al*. [64]	N.d.	↑ Liu *et al*. [73]

^a^ Only nontreated EoE and GERD presented.

Microbiota studies in the colon mainly analyzed fecal samples that may not represent the composition of upper parts of the colon or the terminal ileum. Taking biopsies from the ileum requires colon cleansing, which tremendously affects the composition of the microbiota. Similar limitations should also be considered for the esophagus as taking biopsies in the esophagus by esophagogastroscopy requires a fasting period of four to six hours before the examination. Novel devices for taking esophageal swabs may allow the analysis of the esophageal microbiome in future without a fasting period before the examination. We want to stress that a firm definition of a ‘healthy microbiota’ is lacking. In inflammatory bowel disease, the colon has an increased bacterial load with a simultaneous decrease of diversity, a condition termed ‘dysbiosis’ [69, 70]. However, the term ‘dysbiosis’ lacks a precise definition. ‘Altered microbiota’ provides a better description of the observed changes in the microbiota composition in diseases. In the esophagus, patients with EoE or esophageal adenocarcinomas (EAC) have an altered microbiota [65, 71]. In acid‐induced esophagitis or Barrett's esophagus, Yang *et al*. reported an enrichment in gram‐negative bacteria (anaerobes and microaerophiles) together with an increase in bacterial diversity [65, 72]. Other reports describe an increase in gram‐negative bacteria (e.g., *Veillonella*, *Prevotella*, *Campylobacter*, *Fusobacterium*, *Haemophilus*, *Corynebacterium,* and *Neisseria*) in EoE or acid‐induced esophagitis, further indicating an increased abundance of gram‐negative bacteria in esophagitis [65, 72, 73, 74].

Unresolved questions from these studies include how and which environmental factors, such as diet or drugs, influence the esophageal microbiome. For example, a low fiber diet is associated with expanding mucus degrading bacteria in the colon [75], whereas a fiber‐rich diet reduces mucus degrading bacteria [76]. Simultaneously, a fiber‐rich diet expands bacterial strains that degrade fibers into short‐chain fatty acids (butyrate, acetate, propionate). Short‐chain fatty acids have a vast range of different effects on endogenous metabolism and inflammation, such as satiety regulation [77], browning of white adipose tissue and fat accumulation [78], increased glucagon‐like peptide 1, and peptide YY secretion, and intestinal gluconeogenesis [79, 80]. Only limited data on the effects of diet on the esophageal microbiome exist [81]. One study in pediatric EoE patients investigating the impact of a six‐food elimination diet that avoids wheat, milk, soy, nuts, eggs, and seafood/shellfish on the esophageal microbiome did not reveal any changes the microbiome. After the reintroduction of allergenic foods, *Granulicatella* and *Campylobacter* increased in EoE [65].

The intake of antibiotics also tremendously influences the composition of the microbiome in the GIT. The widespread prescription of antibiotics and agricultural business use, which we are exposed to by drinking water and eating food products, was established in the 1940s [81, 82, 83]. As an unwanted side effect, antibiotic treatment may reduce the microbiome's diversity and disrupt beneficial microbial communities. Potential pathogens may settle in the emerging niches [84]. In the colon, the increased incidence of pseudomembranous enterocolitis caused by *Clostridium difficile* toxin A or B is one of the most significant examples [85]. The intestinal microbiota's reconstitution by fecal microbiota transplant can treat pseudomembranous enterocolitis [86, 87, 88]. In an experimental model of Barrett's esophagus in rats, the animals' treatment with antibiotics did not influence Barrett's esophagus [89]. Interestingly, it has been proposed that the eradication of *Helicobacter pylori* with antibiotics inversely correlates with the development of EAC [90].

Furthermore, PPIs increase the gastric pH by inhibiting acid secretion in the stomach, which may also indirectly affect the esophageal microbiota [81, 91]. PPI treatment increases the Firmicutes phylum members' abundance and decreases the Proteobacteria phylum members' presence in the esophagus [91]. Other widely used drugs that might affect the microbiota composition include nonsteroidal anti‐inflammatory drugs and probiotics [92].Altogether, a unique microbiome distinct from the oral cavity microbiome populates the esophagus. First studies indicate that esophagitis leads to an altered microbiome.

## Eosinophilic esophagitis

**Fig. 1:**
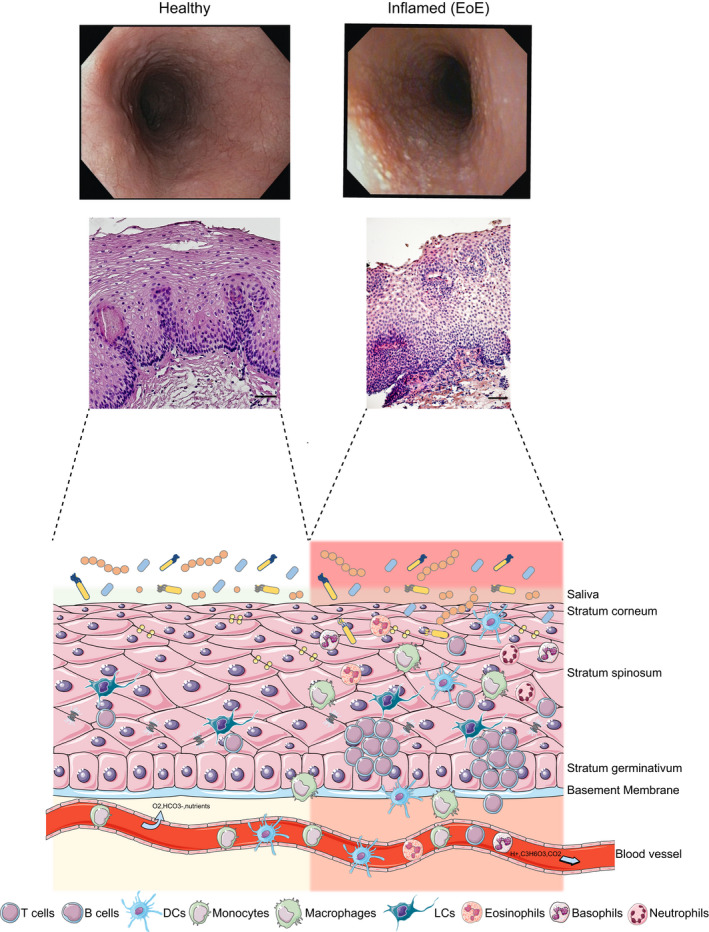
Immune cell composition of the esophagus and in EoE. Macroscopic endoscopic view on the esophagus of healthy individuals and in EoE. In EoE, micro‐abscesses resembling the eosinophil infiltration in the esophagus appear. Histology confirms eosinophil infiltration in the mucosa of EoE. A few immune cells, such as LCs and T cells, are present in the noninflamed esophagus. In EoE, an infiltration of the mucosa with DCs, monocytes, macrophages, eosinophils, basophils, neutrophils, T cells, and B cells occurs. The figure was generated with SERVIER MEDICAL ART 3.0 (The Servier Group, Suresnes, France).

Activation of the immune system associated with an altered microbiome can induce esophageal diseases (Fig. 1). Early antibiotics and PPI in infancy, cesarean delivery, maternal fever, and preterm labor predispose to EoE [93]. EoE is a food‐triggered Th2‐mediated chronic inflammatory disease characterized by eosinophil infiltration (> 15 eosinophils per hpf), increased T and mast cell numbers [4], and associated with an altered microbiome [74]. EoE affects males predominantly compared to females, with a ratio of 3 : 1 [94]. In contrast to the healthy esophagus, which is devoid of eosinophils, the accumulation of eosinophils, attracted by chemokines eotaxin‐1, eotaxin‐3, and cytokines, such as IL‐5 and IL‐13, in the stratum corneum of the esophageal epithelium, characterizes EoE [95, 96, 97, 98]. Eosinophil‐derived granule proteins induce barrier breach and promote a Th2 inflammation [99, 100, 101, 102, 103], resulting in a sustained direct exposure of the esophageal immune system to triggering food allergens leading to transmural inflammation, smooth muscle dysfunction, basal cell hyperplasia, and consequently to fibrosis [104]. Also, mast cells infiltrate the inflamed esophageal tissue to release histamine in EoE [4, 105]. An impaired epithelial barrier has been described in EoE with reduced proteins required to maintain the intact esophageal epithelial barrier, such as filaggrin and desmoglein, combined with dilated intercellular spaces between epithelial cells [5, 6, 106]. Interestingly, genome‐wide association studies suggested genetic risk variants of genes expressed by epithelial and immune cells in EoE [107]. The identified single nucleotide polymorphisms included TSLP [108]; *c11orf30*, *STAT6*, and *ANKRD27* [109]; cytosolic calcium‐activated cysteine proteases *CAPN14* (calpain‐14) [110]; and the filament aggregating protein filaggrin [111]. The cytosolic calcium‐activated cysteine proteases, including CAPN14, modulate integrin‐cytoskeletal interactions, and *filaggrin* binds keratin intermediate filaments, reinforce a barrier dysfunction in EoE. Preclinically, experimental work with patient samples and cell lines by Azouz and colleagues indicated that the reduced expression of the serine protease inhibitor, SPINK7 leads to barrier dysfunction [112]. Further research from the same group suggested that SPINK7 restricts the activity of the serine protease kallikrein 5 (KLK5) and that *klk5*‐deficient mice are protected from the development of an ovalbumin (OVA)‐induced EoE mouse model [113]. It is under discussion whether a barrier defect is a prerequisite for sensitizing the esophageal immune system to EoE and requires continuous antigen exposure to drive esophageal inflammation. However, the observed barrier defect in EoE could also be secondary as a consequence of inflammation in the esophagus. One approach to solve this issue is establishing cohorts in the preclinical phase of EoE before disease onset. In individuals with a preclinical phase of EoE, researchers could investigate the expression of proteins involved in establishing an intact esophageal epithelial barrier.

The epithelial barrier breach further induces the expression of the cytokines TSLP, IL‐25, and IL‐33 that, in turn, drive a Th2‐mediated inflammation [4, 114]. The Th2 cytokine IL‐13 increases the expression of the esophagus‐specific protease CAPN14, which degrades desmoglein‐1 required to form cell‐cell junctions [101, 115]. Furthermore, IL‐13 reduces filaggrin expression in atopic dermatitis [116] and contributes to eosinophil chemotaxis by inducing eotaxin‐3 expression [98, 117]. Increased TSLP expression has been reported in patients with active EoE correlated with eosinophil extracellular trap formation [6]. Patients with a gain‐of‐function polymorphism of TSLP have increased basophil numbers in the esophagus [3]. Moreover, the IL‐1 superfamily member IL‐33, which is constitutively expressed in the nucleus and acts as a cytokine by binding to its receptor ST2, is expressed by the esophageal mucosa [118] and by undifferentiated epithelial cells of EoE patients [119]. Basophils express the IL‐33 receptor ST2, and genetic deletion of ST2 prevents inflammation in an OVA‐induced EoE mouse model [120]. Since EoE patients have increased Th2 cytokine expression, the specific targeting of cytokines with monoclonal antibodies is a promising avenue for the treatment of EoE. The human mAb Dupilumab binds the IL‐4Rα receptor chain blocking both IL‐4 and IL‐13 signaling. In a multicenter phase II trial, Dupilumab improved dysphagia and eosinophil count at week 10 of treatment [121].

Eosinophilic esophagitis is of allergic etiology, corroborated by a high prevalence of concurrent atopic diseases and remendability by allergen avoidance. However, the frequently observed increased food antigen‐specific IgE levels in EoE do not correlate with the EoE‐triggering allergens [122]. Consistently, EoE patients treated with the anti‐IgE antibody, omalizumab, failed in clinical case series and in prospective, randomized, double‐blind, placebo‐controlled studies to show a significant relieve of symptoms [123, 124]. EoE is preferably associated with antigen‐specific IgG4 antibodies [124], which have neutralizing properties due to their week binding affinity to IgG receptors and low complement activation [125, 126, 127]. The elevated IgG4 concentrations in active EoE decrease during dietary interventions by avoiding possible food allergens present in wheat, milk, soy, nuts, eggs, and seafood/shellfish [126]. Altogether, these data suggest that IgE does not drive the pathophysiology in EoE.

**Fig. 2:**
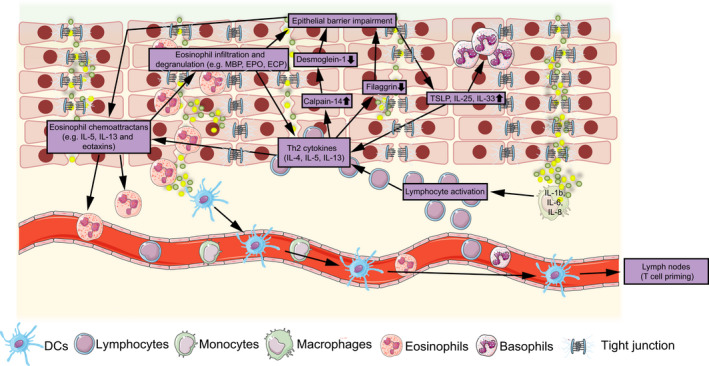
Epithelial barrier impairment and immune cell‐derived cytokines in EoE. In EoE, a breached esophageal barrier is passed by antigens, which are phagocytosed by DCs or macrophages. DCs migrate to draining lymph nodes to prime naïve T cells or activate effector T cells in the lamina propria. T cells produce Th2 cytokines, induce the expression of chemokines, such as eotaxins by epithelial cells for the attraction of eosinophils, and facilitate further barrier breaches by affecting adherens and tight junctions. The graph was designed with SERVIER MEDICAL ART 3.0 (The Servier Group).

In summary, the pathophysiology of EoE is incompletely understood (Fig. 2). Since EoE presents with vomiting and feeding problems in young children and dysphagia and food impaction in adults [128], there is a clinical need for better treatment options. Because food antigens trigger EoE, with milk and wheat as the most prevalent food antigens [129, 130], a six‐food elimination diet with avoidance of wheat, milk, soy, nuts, eggs, and seafood/shellfish is an effective treatment. However, the six‐food elimination diet significantly impacts life quality, limiting the compliance for this treatment for EoE [130, 131, 132]. Topical corticosteroids improve clinical symptoms and are highly efficacious for induction and maintenance therapy of EoE [133, 134]. Small molecules and biologicals that specifically target checkpoints are of significant interest to further improve the treatment of EoE. In the search for targeted therapies of EoE, one has to consider that the inflammation in EoE extends eosinophils' biology. Infiltrating T cells, B cells, and mast cells could provide specific targets for the treatment of EoE [4].

## Conclusions

Increasing evidence indicates that the esophagus is a transport organ with critical importance for mucosal immunity and contributes to immune‐mediated diseases. A better characterization of the esophageal immune system and its relationship with the microbiota will give insights into the development of esophageal diseases. Likely, this research will pave the way for discovering targeted therapies to improve the treatment of esophagitis. We anticipate the following research questions that may be solved before this exciting research will enter daily practice in the clinic.

1. Information on the distribution of immune cells in the healthy esophagus, GERD, and EoE is scarce. Since sampling of esophageal tissues during endoscopy is possible, systematic analysis of immune cells in esophageal biopsies with single‐cell RNA sequencing and mass cytometry will help characterize the cellular composition of the esophageal immune system. These analyses are a requirement before possible targeted therapies in EoE can be explored.
2. Emerging evidence indicates that the esophagus has a core microbiome distinguishable from the oral cavity's microbiome, the skin, the small intestine, and colon. Most assumptions on the esophageal microbiome stem from studies investigating the oral or the intestinal microbiome. There is a need to better characterize the esophageal microbiome in response to the diet, medications, and diseases, such as EoE.

With advances in this area of research, it will in future be possible to move the fascinating research on the esophageal mucosal immunity for the development of targeted therapies for EoE into the clinic. We expect that studies investigating the esophageal mucosal immune system will move into focus soon.

## Conflict of interest

The authors declare no conflict of interest.

## Author contribution

TK and JHN jointly wrote the manuscript, discussed the manuscript with PH, and designed the figures.
